# Mutations of the domain forming the dimeric interface of the ArdA protein affect dimerization and antimodification activity but not antirestriction activity

**DOI:** 10.1111/febs.12467

**Published:** 2013-09-02

**Authors:** Gareth A Roberts, Kai Chen, Edward K M Bower, Julia Madrzak, Arcadia Woods, Amy M Barker, Laurie P Cooper, John H White, Garry W Blakely, Iain Manfield, David T F Dryden

**Affiliations:** 1 EaStCHEM School of Chemistry, University of EdinburghUK; 2 Centre for Biomolecular Interactions Astbury Centre for Structural Molecular Biology, Faculty of Biological Sciences, University of LeedsUK; 3 Institute of Cell Biology School of Biological Sciences, University of EdinburghUK

**Keywords:** antirestriction, ArdA, horizontal gene transfer, restriction enzyme, Tn*916*

## Abstract

ArdA antirestriction proteins are encoded by genes present in many conjugative plasmids and transposons within bacterial genomes. Antirestriction is the ability to prevent cleavage of foreign incoming DNA by restriction-modification (RM) systems. Antimodification, the ability to inhibit modification by the RM system, can also be observed with some antirestriction proteins. As these mobile genetic elements can transfer antibiotic resistance genes, the ArdA proteins assist their spread. The consequence of antirestriction is therefore the enhanced dissemination of mobile genetic elements. ArdA proteins cause antirestriction by mimicking the DNA structure bound by Type I RM enzymes. The crystal structure of ArdA showed it to be a dimeric protein with a highly elongated curved cylindrical shape [McMahon SA *et al*. (2009) *Nucleic Acids Res***37**, 4887–4897]. Each monomer has three domains covered with negatively charged side chains and a very small interface with the other monomer. We investigated the role of the domain forming the dimer interface for ArdA activity via site-directed mutagenesis. The antirestriction activity of ArdA was maintained when up to seven mutations per monomer were made or the interface was disrupted such that the protein could only exist as a monomer. The antimodification activity of ArdA was lost upon mutation of this domain. The ability of the monomeric form of ArdA to function in antirestriction suggests, first, that it can bind independently to the restriction subunit or the modification subunits of the RM enzyme, and second, that the many ArdA homologues with long amino acid extensions, present in sequence databases, may be active in antirestriction.

**Structured digital abstract:**

1. ArdA and ArdA bind by molecular sieving (1, 2)
2. ArdA and ArdA bind by cosedimentation in solution (1, 2)

## Introduction

The majority of eubacteria contain the genes for active or putative DNA restriction-modification (RM) systems 1,2. It is clear that their function is to protect the host cell from invasion by foreign DNA by recognising specific DNA sequences and triggering an endonuclease activity that rapidly cleaves the foreign DNA. The host DNA sequences are maintained in a methylated state by the modification methytransferase (MTase) function.

Despite the demonstrated efficacy of the RM systems, genome analysis of pathogenic bacteria from both clinical and environmental settings makes it abundantly clear that horizontal gene transfer (HGT) by transformation, transduction or conjugation is common within species and even between species. HGT is directly responsible for the spread of antibiotic resistance genes 4. In fact, the spread of antibiotic resistance and the resultant selective pressure caused by the continual use of antibiotics is a serious and almost unstoppable phenomenon 5–8. It is therefore important for understanding and tackling antibiotic resistance to ascertain the mechanism by which HGT circumvents such an apparently effective RM defence. The identification of potential antirestriction and antimodification (anti-RM) genes within the mobile elements 9–10 suggests a mechanism by which the mobile elements can overcome the RM systems. These anti-RM systems have occasionally been acquired and maintained by the host organism, and the occasional activation of such genes weakens or negates the RM defence system, allowing further HGT 11–12.

The first plasmid-borne antirestriction system identified was encoded by the *ardA* locus of the ColIbP-9 enterobacterial plasmids 13. Conserved *ardA* genes have subsequently been identified in representatives of other plasmid incompatibility groups 10–16, other bacterial genomes 17, and ORF18 of the Tn*916* conjugative transposon from *Streptococcus faecalis* 17.

The structure of ORF18 ArdA from Tn*916* reveals a highly elongated dimeric protein with a surface decorated with negative charges in such a way that it mimics the shape and charge distribution of ∼ 42 bp of DNA 18. Thus, ArdA is a DNA mimic anti-RM protein similar to the Ocr DNA mimic anti-RM protein encoded by bacteriophage T7 19,20, although their secondary structures are very different. ArdA monomers are further divided into three small domains composed of amino acids 1–61, 62–103, and 104–165, with the third domain in each monomer forming the dimer interface. The negative charges on the surface of ArdA are spread over all three domains. ORF18 ArdA appears to be able to dissociate into monomers at low concentrations in buffer solution 17, raising the possibility that the monomer form may be active in addition to the dimer form. It may even be the case that one form targets the modification activity and the other form targets the restriction activity of the RM system, as some ArdA proteins show differential effects on restriction and modification, depending on the level of expression *in vivo* 16–25.

The Type I RM systems are the targets for ArdA, and are widespread in nature 26. Moreover, Type I RM systems play a clear role in HGT, as exemplified by the fact that they are used to define clinical strains of *Staphylococcus aureus* 5–27. Type I RM enzymes 2 are complex hetero-oligomers of two restriction subunits (HsdRs), two methyltransferase subunits (HsdMs), and one DNA sequence specificity subunit (HsdS) (total molecular mass of ∼ 440 kDa). Depending on the methylation status of the DNA substrate, this complex functions as either a restriction endonuclease or an MTase. These enzymes recognise an asymmetric, bipartite sequence (for example, EcoKI recognises AACNNNNNNGTGC), and require ATP to affect cleavage at a distant site reached via extensive DNA translocation. Two HsdMs and one HsdS form an active MTase in the absence of HsdR 28–29. Many genomes contain multiple Type I RM systems 3, and some have the ability to switch between multiple DNA specificities 30. Type I RM systems are extensively represented within clinical strain collections such as the *Escherichia coli* ECOR collection 26, and can be grouped into families, defined by subunit complementation for example, in which HsdR and HsdM are highly conserved 31–32. HsdS sequences show extreme variability in two ∼ 150-residue regions. These regions are called target recognition domains (TRDs). The N-terminal TRD recognises the first part of the bipartite sequence, and the C-terminal TRD recognises the second part. TRDs can be swapped within a family to generate predictable changes in the enzyme specificity.

In this study, we investigated the effect of mutagenesis in domain 3 of ORF18 ArdA, which forms the dimer interface and is predicted to interact with the MTase core of a Type I RM enzyme 18. We observed that some of the mutations created solely monomeric forms of ArdA, whereas others either had no effect on protein structure or could not be stably expressed. The purified ArdA proteins, whether monomeric or dimeric, showed reduced antimodification activity against EcoKI, but most retained normal antirestriction activity. These data indicate that antirestriction activity resides in domains 1 and 2 of ArdA, and that antimodification activity resides in domain 3.

## Results

### Location of amino acid substitutions on the dimer of ORF18 ArdA

**Fig 1:**
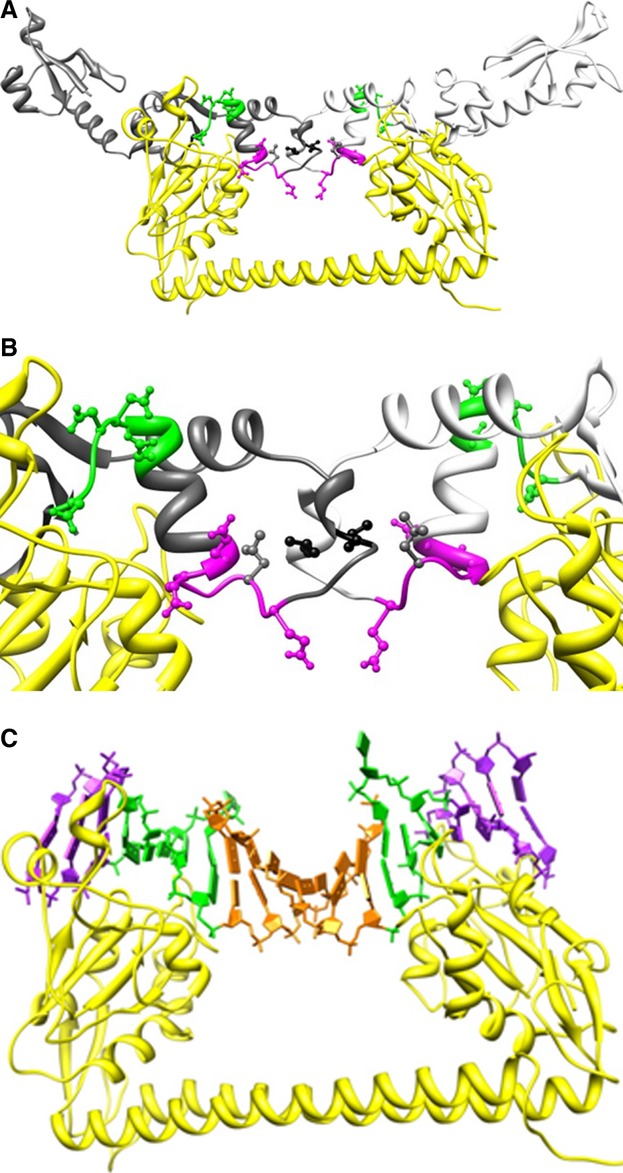
Structural models of the S subunit (yellow ribbon) of EcoKI bound to WT ORF18 ArdA (Protein Data Bank: 2W82) and the DNA target sequence. (A) ArdA chains are shown as grey and white ribbons, with Mut5 and Mut6 regions shown in green and magenta respectively. Sites of amino acid substitution within these regions are shown in a ball and stick representation in green (Mut5), magenta (Mut6), and grey (L127E), respectively. Leu134 is shown in black ball and stick form. (B) An expanded view of the ArdA dimer interface coloured as in (A). (C) The DNA bases are coloured in purple for sequence outside the DNA target sequence, green for the defined bases in the target sequence, and orange for the central undefined bases in the target sequence.

The negatively charged amino acids selected for mutagenesis are shown in Table 1, and were created by the mutagenesis primers Table S1, with plasmid pORF18wt (Fig. S1) as a template. In addition, two leucines (Leu127 or Leu134) at the dimer interface in the crystal structure were individually mutated to glutamate, with the idea that the introduction of a negative charge would prevent formation of the hydrophobic dimer interface. The model of ArdA bound to the EcoKI MTase suggests that these amino acid substitutions occur at positions equivalent to the region of DNA recognised by the S subunit of the RM enzyme (Fig. 1). The physical effects of these mutations on the protein structure were first analysed *in vitro*, to determine whether the substitutions had disrupted the interface to form the desired monomeric forms of ArdA or led to other structural changes. Subsequently, *in vivo* activity tests were performed to determine whether anti-RM activity was affected.

**Table 1:** Amino acid substitutions created in domain 3 of WT ORF18 ArdA

Mutant name	Amino acid changes	Total number of mutated residues
Mut5	D109N, D111N, D112N, D115N	4
Mut6	E122Q, E123Q, E129Q	3
Mut5/6	D109N, D111N, D112N, D115N, E122Q, E123Q, E129Q	7
L127E	L127E	1
L134E	L134E	1

### Characterization of ArdA mutant proteins *in vitro*

Overexpression of the mutant proteins by using mutated forms of plasmid pORF18wt (Fig. S2) was only observed for the Mut5, Mut6 and L127E ArdA proteins, as well as wild-type (WT) ArdA. The other mutant proteins, Mut5/6 and L134E, could not be observed in cell extracts, and nor were either of these proteins observed after fractionation of the cell extracts via ion exchange chromatography (no band of an appropriate size was visible on an SDS/PAGE gel; data not shown). Thus, *in vitro* characterization was confined to Mut5, Mut6 or L127E ArdA. Cells harbouring the various constructs were grown and harvested, and the recombinant proteins were purified to near homogeneity as described previously for WT ORF18 ArdA 17. Figure S2a shows the protein fractions eluting from the anion exchange (DEAE) column prior to further purification by preparative size exclusion chromatography (SEC).

The folding and unfolding curves measured by tryptophan fluorescence were essentially identical for WT ORF18 ArdA 17, and Mut5, Mut6, and L127E ArdA (Fig. S2b). The midpoints of the unfolding transitions were 2.20 ± 0.14 m guanidine hydrochloride 24, 2.39 ± 0.46 m guanidine hydrochloride, 2.44 ± 0.08 m guanidine hydrochloride, and 2.13 ± 0.13 m guanidine hydrochloride, respectively. The free energies of stabilization were 20.0 ± 3.3 kJ·mol^−1^ 17, 15.4 ± 3.4 kJ·mol^−1^, 21.1 ± 4.7 kJ·mol^−1^, and 20.8 ± 4.1 kJ·mol^−1^ respectively. The transition slopes divided by *RT* (ideal gas constant multiplied by temperature) were 1.97 ± 0.33, 1.90 ± 0.47, 3.67 ± 0.77, and 2.03 ± 0.42, respectively. The transition slopes are related to the change in exposed surface area as the protein unfolds. This similarity in stability was expected, as the tryptophans are not located near to the dimer interface, and would only be sensitive to changes in tertiary structure rather than in quaternary structure. CD spectroscopy was used to establish the secondary structure content of all of the purified proteins, and LC-MS was used to determine the exact molecular mass of the Mut5 and Mut6 monomers (Figs S3 and S4). These data suggest that the polypeptide fold was not greatly compromised by the amino acid substitutions.

### Analytical SEC of ArdA

**Fig 2:**
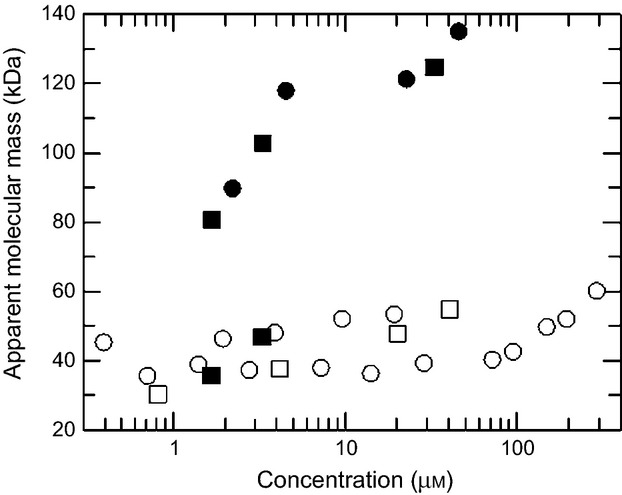
SEC analysis to investigate the solution apparent molecular mass as a function of protein monomer concentration injected onto the column. •, WT ORF18 ArdA; ○, L127E ArdA; □, Mut5 ArdA; ▪, Mut6 ArdA.

It has previously been observed that the apparent molecular mass of WT ORF18 ArdA changes as its concentration changes 17. At high concentration, WT ORF18 ArdA eluted from a SEC column at an apparent molecular mass of > 100 kDa, and at low concentration it eluted with an apparent molecular mass of ∼ 40 kDa (Figs 2 and S5). As the crystal structure 18 showed a dimeric protein, it was assumed that the protein was a dimer of mass 38 kDa at high concentration and a monomer of mass 19 kDa at low concentration, despite the fact that the SEC gave apparent molecular masses far different from these expected values. The discrepancy between observed and expected molecular mass was attributed to the highly elongated shape of the protein, which would give it an unusually large hydrodynamic radius. With this assumption, the data were previously modelled as a monomer–dimer equilibrium with a dissociation constant of 1.3 ± 0.3 μm 17, although this calculation used the concentration of the injected sample rather than the concentration on the column, which will be lower because of dilution during the chromatography.

Mut5, Mut6 and L127E ArdA were also analysed by SEC. Figure 2 (and Figs S5c and S5e) shows that Mut5 and L127E ArdA eluted with an apparent molecular mass of ∼ 40 kDa at all concentrations below 100 μm. Above 100 μm, the apparent molecular mass of L127E ArdA started to increase. The concentrations of the proteins are the concentrations of the injected 40- L samples, and the actual concentration of the samples as they move through the column will be somewhat smaller, owing to sample dilution. If the monomer–dimer equilibrium model for ArdA is correct, then it would appear that Mut5 and L127E ArdA are monomers at most concentrations. Mut6 ArdA behaved in the same manner as WT ORF18 ArdA, showing a decrease in apparent molecular mass as concentration decreased (Figs 2 and S5d).

In order to investigate the discrepancy between the observed and expected molecular masses, an absolute measure of molecular mass was sought for WT ORF18 ArdA and L127E ArdA, by the use of analytical ultracentrifugation (AUC). Such measurements would serve to explain the anomalous molecular masses observed with SEC.

### Sedimentation equilibrium AUC of ArdA

**Fig 3:**
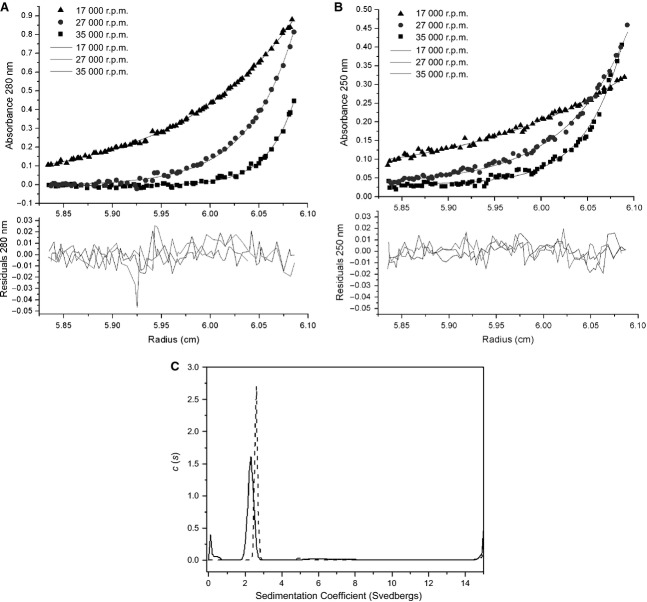
AUC of WT ORF18 ArdA and L127E ArdA. (A) sedphat sedimentation equilibrium data analysis of WT ORF18 ArdA at 15 μm at 17 000 r.p.m., 27 000 r.p.m., and 35 000 r.p.m., with detection at 280 nm. The samples had reached equilibrium at each rotor speed, as the rmsd between scans was below ± 0.01 absorbance units (typical noise level in the centrifuge). The fitted line and residuals are for a single-species fit. (B) sedphat sedimentation equilibrium data analysis of L127E ArdA at 15 μm at 17 000 r.p.m., 27 000 r.p.m., and 35 000 r.p.m., with detection at 280 nm. The samples had reached equilibrium at each rotor speed, as the rmsd between scans was below ± 0.01 absorbance units (typical noise level in the centrifuge). The fitted lines and residuals are for a two-species fit. (C) sedfit sedimentation velocity *c*(*s*) distributions of WT ORF18 ArdA (dashed line) and L127E ArdA (solid line) show that the proteins have different sedimentation velocity properties in solution.

Sedimentation equilibrium measurements can give an absolute value for molecular mass. Three different sample concentrations of WT ArdA or L127E ArdA were analysed individually, to give an idea of their behaviour in solution (Fig. 3A,B). Initially, all samples were modelled as single species (Table 2). The whole cell weight average molecular mass of WT ORF18 ArdA was not observed to change over the concentration range studied (0.6–15 μm), and had an average of 37.4 kDa, indicating that WT ORF18 ArdA exists as a dimer (Fig. 3A). The detection limits on the AUC instrument precluded measurements of the dissociation of WT ORF18 ArdA at very low concentrations. A global analysis for WT ORF18 ArdA was also performed, assuming the same molecular mass at all three concentrations, to confirm that a single average molecular mass was appropriate. Individual analyses for L127E ArdA showed that the molecular mass changed with respect to concentration and that the global analysis was inappropriate (Table 2). At low concentrations, the molecular mass was between 21.2 and 17.0 kDa, indicating that it is a monomer in solution at these concentrations (Table 2). However, L127E ArdA at 15 μm was modelled as two species, and the fit was improved (Table 2; Fig. 3B). The two species in this fit had molecular masses of 39.1 and 20.3 kDa, suggesting that, at 15 μm, L127E ArdA is in rapid exchange between monomeric and dimeric forms. The presence of monomers and dimers at high concentration is consistent with the increase in the apparent molecular mass for L127E ArdA observed in the SEC experiment at high concentrations (Fig. 2). The 15 μm data were also modelled as a monomer–dimer equilibrium with sedphat 33, fixing the monomer molecular mass of 19.1 kDa. This gave a dissociation constant of 10 μm, but carried a high rmsd of 0.01 (a good fit has 0.001) and largely reduced *χ*^2^ value of 7.9 (a good fit has a value of 1), suggesting a poorer fit than the two-species fit, which had a *χ*^2^ of 1.95.

**Table 2:** Sedimentation equilibrium analytical ultracentrifugation analysis for determination of molecular mass (*m*) in kDa. Rmsd and reduced *χ^2^* are given

	15.0 μm			3.0 μm			0.6 μm			Global analysis of all concentrations		
Sample	*m*	rmsd	*χ*^2^	*m*	rmsd	*χ*^2^	*m*	rmsd	*χ*^2^	*m*	rmsd	*χ*^2^
WT ORF18 ArdA	39.4	0.010	4.34	36.2	0.014	7.35	38.3	0.014	8.19	37.4	0.010	6.36
L127E ArdA	27.3^a^	0.007	2.12	21.2	0.005	1.03	17.0	0.005	1.15	26.7	0.010	2.37

a The value given is for analysis assuming a single species. However, at the highest concentration, the data fitted best to two species in solution with molecular masses of 39.1 and 20.3 kDa (rmsd = 0.007, *χ*^2^ = 1.95). Global simultaneous analysis of all three concentrations assuming a single species was performed with sedphat.

The discrepancy in the concentration range between the SEC and the AUC experiments in which the two proteins show monomeric–dimeric behaviour can be explained by the fact that the SEC column dilutes the protein by approximately five-fold to 10-fold but we have reported the injected SEC protein concentration and calculated the apparent dissociation constant for the proteins by using the injected concentration. As the SEC detection was performed with tryptophan fluorescence emission, there is no reliable method to determine the dilution factor.

### Sedimentation velocity AUC of ORF18 ArdA and L127E ArdA

The samples for sedimentation velocity AUC had concentrations of 33.9 μm and 36.9 μm, so WT ORF18 ArdA should be primarily in the large molecular mass form and L127E ArdA in both the monomeric and dimeric forms, as judged from the SEC data shown in Fig. 2 and the sedimentation equilibrium results. Absorbance scans before and after a low-speed centrifugation step were identical, indicating the absence of high molecular mass aggregates. Preliminary absorbance scans of the samples indicated that the absorbance at 280 nm would be outside of the linear range of the detector, but that 260 nm would give an acceptable signal.

Analysis of the radial absorbance scans (Fig. S6) used continuous *c*(*s*) distribution analysis to show the distribution of sedimentation coefficients in the samples (Fig. 3C). For the major species present in each sample, calculated molecular masses and proportions are summarized in Table 3. The major species (89%) for WT ORF18 ArdA had a sedimentation coefficient, *s*^o^_20,w_, of 2.6 S, and the major species (78%) for L127E ArdA had an *s*^o^_20,w_ of 2.3 S. Other minor species contributed to the remaining signal, particularly for L127E ArdA. These small amounts of material had *s*-values between 15 S and 50 S in the *c*(*s*) analysis (data not shown). L127E ArdA showed a lower sedimentation coefficient than WT ORF18 ArdA and a slightly broadened peak. The lower sedimentation coefficient could be attributable to increased asymmetry, unfolded protein conformations, a change in protein hydration, or a different multimeric state. The broadened peak could arise from equilibrium between a monomeric L127E ArdA and a dimeric form. Assuming that a different multimeric state is the source of the difference in sedimentation coefficient between WT ORF18 ArdA and L127E ArdA, sedfit 34 designated apparent molecular masses for the two species as 32.6 kDa for WT ORF18 ArdA and 15.1 kDa for L127E ArdA. The *f*/*f*_o_ determined from data analysis was also examined.

**Table 3:** Estimated molecular mass distributions and *f*/*f*_o_ values from sedimentation velocity AUC. Rmsd values of sedimentation coefficient (*s*^o^_20,w_) and *f*/*f*_o_ are given for the major sedimenting species. The rmsd at a confidence level of 0.683 (one standard deviation) is given for the major sedimenting species

Sample	Concentration (μm)	Measured sedimentation coefficient (*s*^o^_20,w_) (% composition) of 260-nm signal			Total%	rmsd	*f*/*f*_o_
WT ORF18 ArdA	33.9	2.6 (89.0)	5.2 (4.3)	9.0 (3.0)	96.3	0.0076	1.50
L127E ArdA	36.9	0–0.25 (6.3)	0.25–0.75 (2.6)	2.3 (77.7)	86.6	0.0100	1.08

The frictional ratio (*f*/*f*_o_) is given in Table 3. This is a parameter describing the asymmetry of the molecules sedimenting in solution. A spherical unhydrated molecule will have an *f*/*f*_o_ of 1.0, whereas values in the range 1.1–1.3 are expected for hydrated, globular proteins 35. This *f*/*f*_o_ analysis suggests that WT ORF18 ArdA with an *f*/*f*_o_ of 1.50 is very asymmetric, as would be expected from the crystal structure. Assuming a hydration level of ∼ 30% for the proteins, the experimental *f*/*f*_o_ for WT ORF18 ArdA gives an axial ratio of ∼ 7 if modelled as a prolate ellipsoid 35.

The data indicate that L127E ORF18 ArdA has an *f*/*f*_o_ of 1.08 (Table 3), which would correspond to an almost globular protein if there were only a single species present in solution. The crystal structure of ArdA indicates that a monomer of ArdA should have an axial ratio of ∼ 3.5 if modelled as a prolate ellipsoid, and an *f*/*f*_o_ of ∼ 1.25. The observed *f*/*f*_o_ for L127E ArdA was lower than expected, but this can be explained by the measurement of sedimentation velocity being skewed by the presence of more than one species, namely the monomer–dimer equilibrium indicated by SEC and sedimentation equilibrium.

Furthermore, calculation of sedimentation velocity and *f*/*f*_o_ by hydropro and somo 36,37 from bead models calculated with the crystal monomer and dimer showed good agreement with the experimental data for WT ORF18 ArdA, and, because of the monomer–dimer equilibrium, only qualitative agreement for the mutant (Table 4).

**Table 4:** Comparison of experimental sedimentation velocity and *f*/*f*_o_ for the major sedimenting species, with values calculated with somo 37–38 and hydropro 36, based on the crystal structure of the ArdA dimer for WT ORF18 ArdA 18 and the monomer coordinates extracted from the crystal structure for L127E ArdA

	Sedimentation velocity		somo		hydropro	
Protein	*s*^o^_20,w_	*f/f*_o_	*s*^o^_20,w_	*f*/*f*_o_	*s*^o^_20,w_	*f*/*f*_o_
WT ORF18 ArdA	2.6	1.50	2.64	1.55	2.40	1.63
L127E ArdA	2.3	1.08	1.92	1.35	1.72	1.43

### Comparison of *in vivo* activity of ORF18 ArdA and ArdA mutants against the EcoKI Type I RM system

After determination of the effects of the amino acid substitutions on the quaternary structure of ArdA, the ability of the mutant ArdA proteins to inhibit restriction and modification by the EcoKI RM system *in vivo* was examined. The *in vivo* activities of WT ORF18 ArdA and the mutant ArdA proteins were examined with a restriction assay in which the ability of phage λv.0 to infect two strains of *E. coli*, one with the EcoKI RM system, *E. coli* NM1049(DE3), and one without the RM system, *E. coli* NM1261(DE3), transformed with the expression plasmids, was investigated (Table 5). The control experiment with the pTRC99 vector 39 showed that *E. coli* NM1261(DE3) was easily infected by phage λv.0 (high titre), but that *E. coli* NM1049(DE3) was not easily infected (low titre), owing to restriction by the EcoKI RM system.

**Table 5:** *In vivo* anti-RM activity of ArdA proteins. The titre of phage per millilitre was determined in *E. coli *NM1261(DE3, r^−^m^−^) and *E. coli* NM1049(DE3, r^+^m^+^), and the ratio was calculated (phage per millilitre in NM1049/phage per millilitre in NM1261) to obtain the efficiency of plating. The two strains were transformed with either the vector alone or plasmids expressing mutants of ArdA

Plasmid name	Efficiency of plating of phage λ	Antirestriction	Antimodification	Significant antimodification
ptrc99a	3.0 × 10^−4^	0.0003	0.9	–
pORF18wt	1.1 × 10^0^	1	122.0	Yes
Mut5	6.0 × 10^−2^	0.054	0.7	No
Mut6	8.5 × 10^−1^	0.773	3.0	No
Mut5/6	3.4 × 10^−3^	0.003	0.9	No
L127E	8.6 × 10^−1^	0.782	1.0	No
L134E	2.3 × 10^−2^	0.021	3.0	No

Cells transformed with plasmid expressing WT ORF18 ArdA or active ArdA mutants (Mut6 and L127E) showed a high titre of phage for both bacterial strains, indicating that the ArdA proteins were abolishing the restriction activity of EcoKI and were ∼ 80% as effective as WT ORF18 ArdA. Cells transformed with plasmids expressing Mut5 were only 5% as active as WT ArdA; L134E ArdA showed 2% activity, and Mut5/6 ArdA showed essentially zero (0.3%) inhibition of restriction by the EcoKI system. The low antirestriction activity of Mut5/6 and L134E ArdA is most probably a consequence of their poor expression, whereas the low activity of Mut5 ArdA, which was expressed well, is suggestive of a defect in activity.

A further *in vivo* test was performed to determine whether some of the mutant ArdA proteins were able to prevent modification of phage λv.0 by EcoKI. Phage were recovered after growth on *E. coli* NM1049(DE3) transformed with the plasmid expressing WT ORF18 ArdA or with plasmid expressing Mut5, Mut6, L127E or L134E ArdA, and tested for modification by comparing the titre of the recovered phage on *E. coli* NM1049(DE3) and *E. coli* NM1261(DE3) (Table 5). An antimodification value of 122 was obtained for phage recovered from the strain transformed with the WT ORF18 ArdA plasmid, indicating that a proportion of the recovered phages were unmodified and that ORF18 ArdA was partially active as an antimodification protein, as expected. However an efficiency of plating of order 1 was obtained for phage recovered from the strains expressing the ArdA mutants or containing the plasmid vector. This indicated that these mutant proteins were not able to prevent the methylation reaction of EcoKI *in vivo*, and that the phage DNA had been modified. Whereas this loss of antimodification activity can be attributed to low protein expression for Mut5/6 and L134E ArdA, the other proteins were expressed well, so the loss of antimodification activity indicates a loss of interaction between the ArdA mutant and the EcoKI enzyme.

## Discussion

Our results show that mutations in domain 3 of ORF18 ArdA affect the ability of the protein to form the dimer observed in the crystal structure. Examination of the structural model of the ArdA dimer bound to HsdS of the EcoKI MTase (Fig. 1A,B) 18 and the model of DNA bound to HsdS (Fig. 1C) reveals that the locations of the mutations analysed in this study are located near the DNA-binding site of the TRDs in HsdS (Mut5 and Mut6) or at the ArdA dimer interface (L127E and L134E). The observation that Mut5 ArdA is a monomer when the substitutions made are not at the interface suggests that they have caused a minor alteration in protein structure, which manages to propagate through the structure to the dimer interface, preventing its correct formation. Of note is the change D112N at the start of an α-helix. Removal of the charge at this end of the helix and consequent interference with the normal electrostatic dipole of the helix may alter its orientation, even though no affect on folding stability or secondary structure was observed. The substitutions forming Mut6 ArdA commence at the other end of this same α-helix, but do not disrupt dimerization. As most of the substitutions in Mut6 ArdA are on a loop in the structure, perhaps there is sufficient flexibility in the loop to accommodate them but not in the helix region in Mut5 ArdA.

The concatenation of Mut5 and Mut6 to make Mut5/6 prevented protein expression, probably because of exacerbation of the folding problem present in Mut5 ArdA. Leu127 and Leu134 are in contact with each other across the dimer interface in the crystal structure, so it was not unexpected that their replacement with a large negatively charged side chain in an already charged region (Leu127 lies within the region mutated in Mut6 ArdA) would disrupt the interface (L127E ArdA) and even lead to expression problems (L134E).

Our recent mutational analysis of the Ocr DNA mimic binding to the EcoKI MTase defined similar regions of interactions with HsdS of EcoKI as delineated here for Mut5 and Mut6 ArdA 40. Thus, despite the completely different folds of Ocr and ArdA, the equivalent regions on their surface are in contact with the EcoKI MTase. The region defined by Mut5 ArdA interacts with part of the TRDs of HsdS, which recognise the specified bases in the EcoKI target, i.e. the AAC and GTGC parts of the target sequence, whereas the region defined by Mut6 ArdA (and Leu127) interacts with the region of HsdS that interacts nonspecifically with the six base pairs in the middle of the bipartite target sequence. Mutations in Ocr affecting DNA binding affinity were clustered in the same location as Mut6 ArdA, and also resulted in a weaker interaction between the Ocr mutant and the EcoKI MTase than between the MTase and its DNA target.

Although the solution environment *in vivo* is very different from the *in vitro* conditions, it appears reasonable to assume that the ability to retain antirestriction activity does not depend on the quaternary structure of ArdA, as one of the monomeric forms, L127E ArdA, retained 80% antirestriction activity, whereas the other monomeric form, Mut5 ArdA, showed only 5% activity. The dimeric Mut6 ArdA retained 80% antirestriction activity. None of these three mutants, Mut5 ArdA, Mut6 ArdA, or L127E ArdA, showed antimodification activity *in vivo*. However, if one again assumes that the *in vitro* results can be extended to the *in vivo* situation, the inability of these mutants to cause antimodification is not attributable to the quaternary structure of ArdA, as both the monomeric and dimeric mutant ArdA forms failed to inhibit modification. Nor can the loss of antimodification activity be attributed to low protein expression levels, as these mutant proteins all expressed well. Our results obtained *in vivo* agree with earlier work 21–25 where *in vivo* expression levels of ArdA from the ColIb-P9 plasmid were varied. These *in vivo* experiments showed that antirestriction was prevalent over antimodification when the concentration of ArdA was low. It was proposed that monomers of ArdA inhibited only the restriction activity, because they could only bind to the HsdRs of Type I RM enzymes, whereas dimers of ArdA could also bind to the MTase core of the RM enzyme 21–25. Such an interaction between HsdR and the Ocr antirestriction protein has been observed previously 41, so it could be expected that ArdA would also have binding sites on the RM enzyme in addition to the binding site on the core MTase. It would be of interest to examine the HsdR–ArdA interactions in more detail but, unfortunately, HsdR of EcoKI and the complete RM enzyme are available in too small amounts for meaningful biophysical analyses. As the structural model suggests that domain 3 of ArdA is primarily responsible for interacting with the MTase core of the RM enzyme (Fig. 1), domains 1 and 2 of ArdA projecting beyond the MTase core would appear to contain the binding surface for HsdR of the RM enzyme, as suggested previously 18.

Finally, a search of the NCBI database using the Tn*916* ORF18 ArdA sequence and blink (http://www.ncbi.nlm.nih.gov/sutils/blink.cgi?mode=query) reveals putative *ardA* genes in many conjugative transposons and prophage integrated into the genomes of a large number of bacterial species. Sequenced *ardA* genes are mostly predicted to encode small polypeptides of 166–177 residues, many of which are highly acidic and carry a net negative charge of − 22 to − 29. A proportion of the putative ArdA proteins in the NCBI database are larger than a typical ArdA, which comprises 166–177 residues. Some of these larger ArdA proteins contain significant N-terminal or C-terminal extensions, indicating that the ArdA monomer has been fused to the end of another protein. A smaller number of putative ArdA proteins appear to have both N-terminal and C-terminal extensions. If these sequences were actually translated into protein, then our data on the monomeric forms of ArdA would indicate that these putative antirestriction proteins could bind to and inhibit the restriction reaction of HsdRs in Type I RM systems in a wide range of organisms.

## Experimental procedures

The *E. coli* strains used and methods for assessing *in vivo* activity of ArdA and its mutants were essentially the same as described previously 40, although only spot tests, rather than full plate assays, were used to determine the antimodification activity. The protocol for analytical SEC has been described previously 40. The buffer for the chromatography was 20 mm Tris, 20 mm Mes, 200 mm NaCl, 10 mm MgCl_2_, 0.1 mm EDTA, and 7 mm 2-mercaptoethanol (2-ME), adjusted to pH 6.5 with HCl. The flow rate was set to 0.5 mL/min, and the sample volume was 40 μL. The column eluate was excited at 295 nm, and the fluorescence emission was continuously monitored at 350 nm.

### Construction of ORF18 ArdA mutants, and protein purification and characterization

The construction of a plasmid containing the ORF18 *ardA* gene from Tn*916* (Fig. S1) and the purification of the protein have been described previously 17–18. Site-directed mutagenesis was carried out with the QuikChange II Site-Directed Mutagenesis kit from Stratagene (La Jolla, CA, USA), with the primers shown in Table S1, to create the substitutions shown in Table 1. DNA sequencing confirmed that the desired changes in the ORF18 *ardA* gene had been achieved.

The ArdA proteins were overexpressed and purified as described previously 17. The extinction coefficient for the monomeric form of ArdA was calculated from the amino acid sequence (WT ORF18 ArdA, 28 020 m^−1^·cm^−1^) and used to calculate the protein concentration. Small differences in extinction coefficient resulting from the mutations were ignored, as the coefficient is only accurate to ± 5% 42. All ArdA concentrations are expressed in terms of monomer concentration.

Equilibrium unfolding as a function of guanidinium chloride was monitored by tryptophan fluorescence spectroscopy. WT ORF18 ArdA has two tryptophans, Trp23 and Trp70, located in domains 1 and 2, distant from the dimer interface. A stock solution of guanidinium chloride was made up, and the precise concentration was determined from the refractive index 43. Protein (3.5 μm) in 20 mm Tris/HCl, 10 mm MgCl_2_ and 7 mm 2-ME (pH 8.0) was incubated with various concentrations of guanidinium chloride at 25 °C, and allowed to equilibrate overnight. The fluorescence intensity was then measured for each sample, with excitation at 295 nm and emission at 350 nm and 380 nm, with 5-nm bandwidths, on an Edinburgh Instruments FS900 fluorimeter (Edinburgh Instruments, Livingston, UK). The ratio of intensity at 350–380 nm was then fitted to a two-state unfolding model assuming a linear relationship between free energy of unfolding and the concentration of guanidinium chloride 44. This ratio compensates for slight differences in the protein concentration between samples.

CD measurements were carried out on a Jasco Model J-180 spectropolarimeter (Jasco Corporation, Tokyo, Japan). All measurements were conducted in 20 mm sodium phosphate, 50 mm NaF, and 0.5 mm 2-ME (pH 8.0). Far-UV CD spectra were measured in the range 190–260 nm at protein concentrations of 5.6–5.8 μm. All CD measurements were made at 20 °C in a 1.0-mm pathlength cell, and each spectrum was the average of three individual scans. The spectra were corrected for buffer contribution.

LC-MS experiments were performed by D. Clarke (School of Chemistry, University of Edinburgh). For LC-MS, an Ultimate 3000 HPLC system (Dionex Corporation, Sunnyvale, CA, USA), equipped with a monolithic PS-DVB (500 μm × 5 mm) analytical column (Dionex Corporation), was used. Samples containing ∼ 1 μg of protein were injected into the column. MS data were acquired on a Bruker 12 Tesla Apex Qe FT-ICR (Bruker Daltonics, Billerica, MA, USA) equipped with an ESI source. Fast Fourier transforms and subsequent analyses were performed with dataanalysis (Bruker Daltonics) software.

### AUC

Prior to any AUC, the samples were analysed with UV absorbance spectrophotometry to determine an appropriate wavelength for data collection. Stock protein samples were subjected to a 3000 r.p.m. (∼500 **g**) spin in a benchtop microcentrifuge to remove any particulates. The buffer used was 200 mm NaCl, 20 mm Tris, 20 mm MES, and 1 mm tris(2-carboxyethyl)phosphine (TCEP), adjusted to pH 6.5 with HCl. TCEP was used instead of 2-ME in the AUC experiments. The density and viscosity were calculated to be *ρ* = 1.00704 g·mL^−1^ and *η* = 1.026 × 10^−2^ Poise, respectively. Owing to software restrictions, the values for density and viscosity omitted the presence of 20 mm MES and 1 mm TCEP. The partial specific volumes (υ-bar) for ORF18 ArdA and L127E ArdA were calculated from their amino acid composition to be 0.726 mL·g^−1^ and 0.725 mL·g^−1^, respectively. The partial specific volume of the sample and buffer density and viscosity were calculated with sednterp V1.09 (March 2006) 45.

For sedimentation equilibrium, we used samples (0.125 mL) centrifuged in 1.2-cm pathlength six-sector epon centrepiece cells with sapphire windows in a four-place An-60 Ti analytical rotor at a temperature of 20 °C. Rotor speeds (17 000, 27 000 and 35 000 r.p.m.) were calculated on the assumption of a monomer molecular mass of 19 125 Da. Because of the range of concentrations present (15 μm, 3 μm and 0.6 μm in monomers), radial absorbance scans at 230, 250 and 280 nm, with 20 scans with a radial step size of 0.001 cm, were performed and interference scans were also performed. Scan intervals at 17 000 r.p.m. (23 305 **g**) were after 12 h, and then every 1 h until equilibrium was reached. Scan intervals at 27 000 (58 787 **g**) and 35 000 r.p.m. (98 784 **g**) were after 9 h, and then every 1 h until equilibrium was reached. Scans were judged to be at equilibrium with sedfit V11.9 (July 2010), by looking at the difference between successive scans 34. Blank water scans were also collected at all speeds and wavelengths used. The results were analysed with sedphat V6.5 (June 2009) 33 (P. Schuck, http://www.analyticalultracentrifugation.com/sedphat/sedphat.htm; and https://sedfitsedphat.nibib.nih.gov/default.aspx). The total run time was just over 68 h. After data collection, the rotor was accelerated to 48 000 r.p.m. (185 795 **g**) to pellet macromolecular material and to measure the baseline absorbance resulting from any low molecular mass material.

For sedimentation velocity AUC, we used 0.406-mL samples centrifuged in 1.2-cm pathlength two-sector aluminium centrepiece cells built with sapphire windows in an eight-place An50 Ti analytical rotor running in an Optima XL-I analytical ultracentrifuge (Beckman Instruments, Fullerton, CA, USA) at 50 000 r.p.m. (201 600 **g**) and a temperature of 20 °C. Changes in solute concentration were detected by radial absorbance scans at 260 nm; 200 scans per cell were collected over 15 h in radial stepwise increments of 0.003 cm, with a scan interval of 2 mins. Results were analysed by whole boundary profile analysis with sedfit V11.9 (July 2010) 34. The fitting resolution in sedfit of the sedimentation velocity AUC data is 150. The concentration of ORF18 ArdA was 33.9 μm, and that of L127E ArdA was 36.9 μm, in terms of monomers. Hydrodynamic parameters were calculated from the crystallographic data with hydropro 36 and somo 37–38.

## Abbreviations

2-ME: 2-mercaptoethanol
anti-RM: antirestriction and antimodification
AUC: analytical ultracentrifugation
*f*/*f*_o_: frictional ratio
HGT: horizontal gene transfer
HsdM: methyltransferase subunit
HsdR: restriction subunit
HsdS: DNA sequence specificity subunit
MTase: methyltransferase
RM: restriction-modification
SEC: size exclusion chromatography
TCEP: tris(2-carboxyethyl)phosphine
TRD: target recognition domain
WT: wild-type

## Supporting information

**Table S1.** Sequence of mutagenic oligonucleotide primer pairs.

**Fig. S1.** Map of pORF18wt.

**Fig. S2.** Protein purification and folding curves.

**Fig. S3.** Circular dichroism spectra.

**Fig. S4.** LC-MS.

**Fig. S5.** Size exclusion chromatography.

**Fig. S6.** Sedimentation velocity scans.
