# Supplementary material for: Mutations of the domain forming the dimeric interface of the ArdA protein affect dimerization and antimodification activity but not antirestriction activity
Source: FEBS J. 2013 Sep 2;280(19):4903–14. doi: 10.1111/febs.12467 (PMC3906837; doi:10.1111/febs.12467)
Supplement: Supplementary file 1 [file febs0280-4903-sd1.pdf]

the FEBS Journal
WILEY Blackwell
www.febsjournal.org

Mutations of the domain forming the dimeric interface of the ArdA protein affect dimerization and antimodification activity but not antirestriction activity

Gareth A. Roberts, Kai Chen, Edward K. M. Bower, Julia Madrzak, Arcadia Woods, Amy M. Barker, Laurie P. Cooper, John H. White, Garry W. Blakely, Iain Manfield and David T. F. Dryden

DOI: 10.1111/febs.12467

Mutations of the domain forming the dimeric interface of the ArdA protein affect dimerisation and antimodification activity but not antirestriction activity.

Gareth A. Roberts[1,+], Kai Chen[1,+], Edward K.M. Bower[1], Julia Madrzak[1], Arcadia Woods[1], Amy M. Barker[2], Laurie P. Cooper[1], John H. White[1], Garry W. Blakely[3], Iain Manfield[2] and David T.F. Dryden[1,*]

Supplementary information

Table S1. Sequence of mutagenic oligonucleotide primer pairs used to generate the desired mutations. Initially, wild-type Orf18 DNA was used as template (pORF18wt, see figure S1). The PCR mix (total volume 50 µl) included *PfuUltra* HF DNA polymerase (2.5 U), 10 mM Tris-HCl (pH 9.0 at room temperature), 50 mM KCl, 1.5 mM $MgCl_2$, 200 µM of each deoxynucleoside triphosphate, 125 ng of each oligonucleotide primer and ~5 ng of template DNA. Temperature cycling for mutagenesis was as follows: 30 s at 95ºC, followed by 18 cycles of 30 s at 95ºC, 1 min at 55ºC, 6.5 min at 68ºC. The generation of Mut5/6 required two separate rounds of mutagenesis i.e., oligonucleotide primers for Mut5 were used to introduce mutations into the construct encoding Mut6 (used as template).

Mutant	Primer type	Primer sequence
**Mut5**	Forward	5′-CATTCATTCCAATTGTAATAATATGTATAACGTGGCACG-3′
	Reverse	5′-CGTGCCACGTTATACATATTATTACAATTGGAATGAATG-3′
**Mut6**	Forward	5′-CTACTACATTCAACAAACGGGTGCTTTAGGCCAAGTACCAGC-3′
	Reverse	5′-GCTGGTACTTGGCCTAAAGCACCCGTTTGTTGAATGTAGTAG-3′
**L127E**	Forward	5′-GAAACGGGTGCTGAAGGCGAAGTACCAG-3′
	Reverse	5′-CTGGTACTTCGCCTTCAGCACCCGTTTC-3′
**L134E**	Forward	5′-GAAGTACCAGCTAGTGAGCAAAACTATATTG-3′
	Reverse	5′-CAATATAGTTTTGCTCACTAGCTGGTACTTC-3′

Supplementary Figure S1. Map of pORF18wt. The gene encoding wt Orf18 ArdA (green) was positioned immediately downstream of the IPTG-inducible promoter (Trc). The plasmid also contains an ampicillin resistance gene as a selection marker.

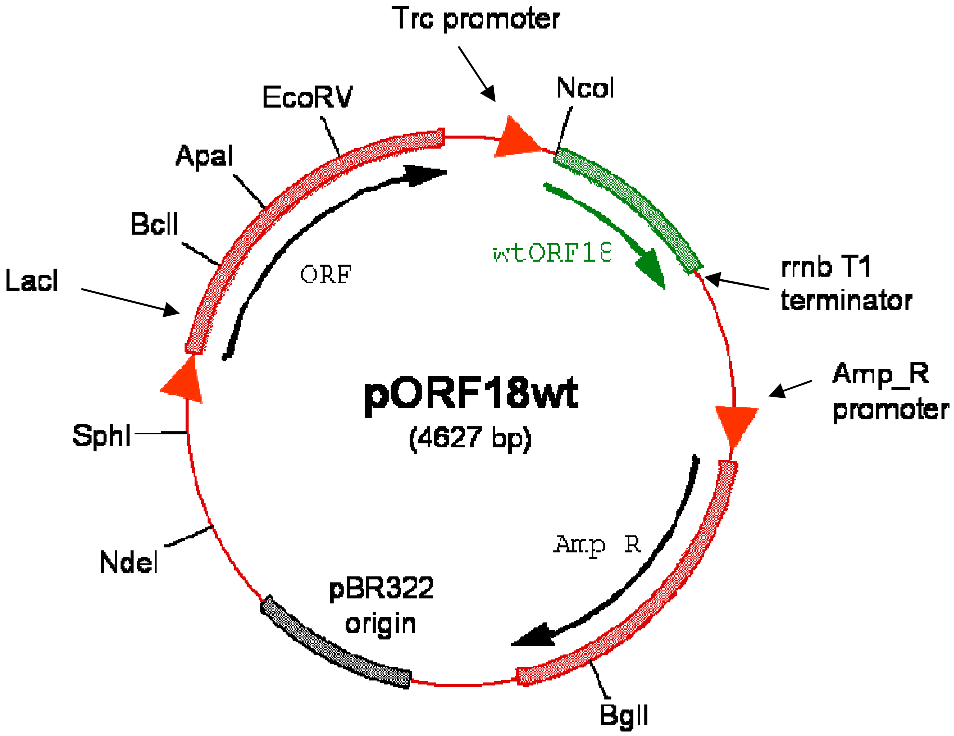

Supplementary Figure S2 (a) Protein purification. Fractions obtained after anion exchange chromatography using a DEAE column. Panel A, L127E ArdA; Panel B, Mut5 ArdA; Panel C, Mut6 ArdA. **(b)** The folding curves measured by tryptophan fluorescence.

(a)

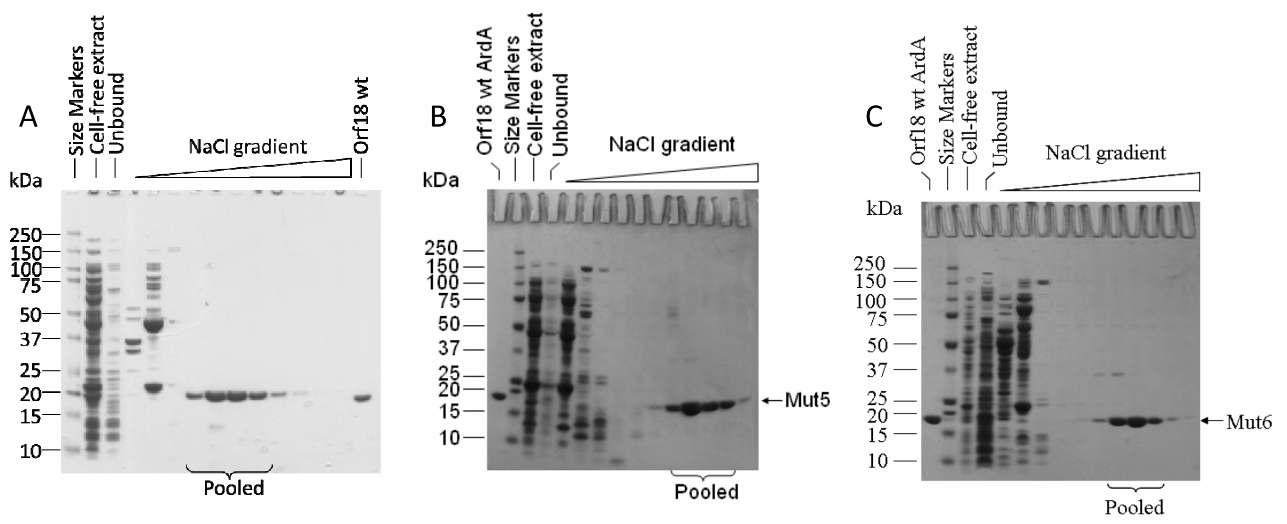

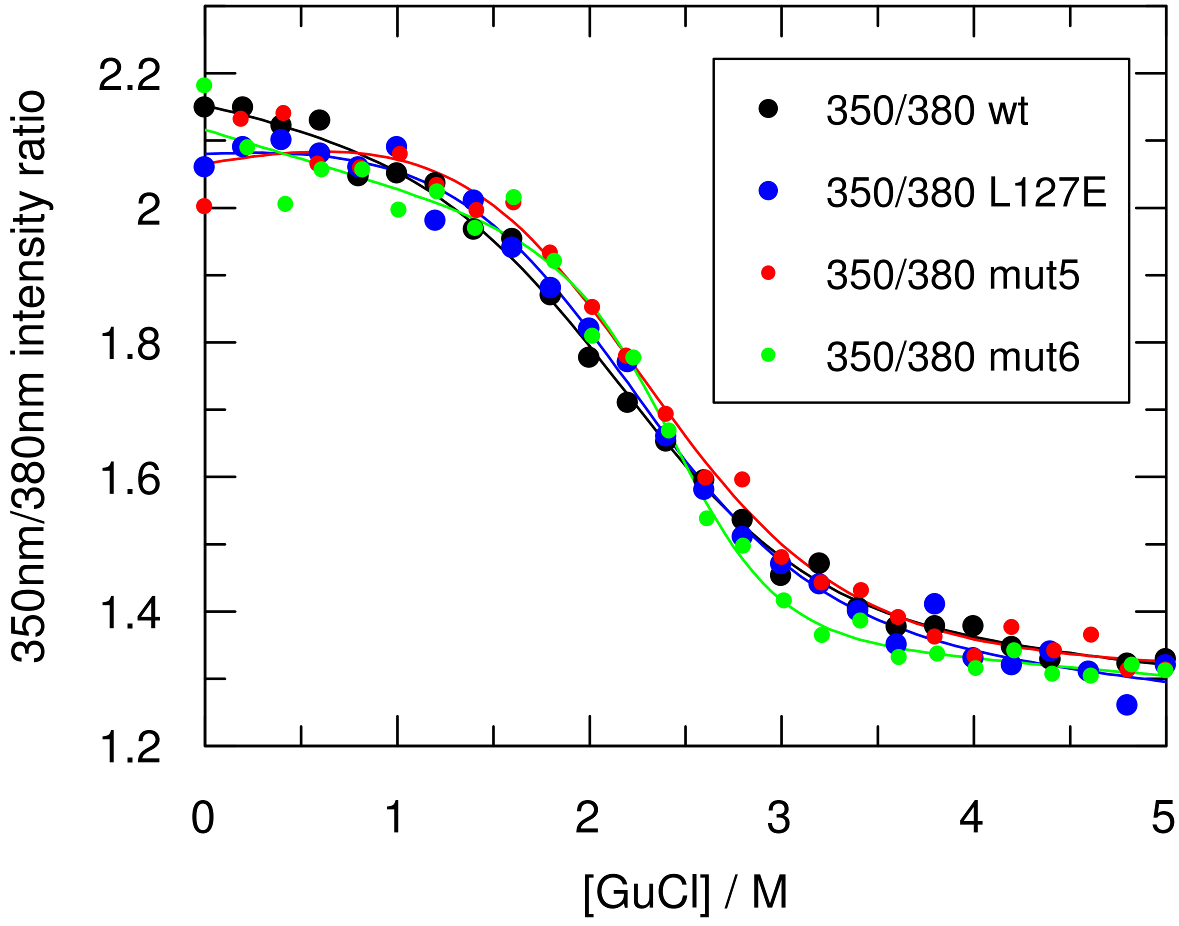

(b)

Supplementary Figure S3. Circular dichroism spectra of wt Orf18 ArdA, Mut6 ArdA, Mut5 ArdA and L127E ArdA. Each protein was buffer exchanged into 20mM sodium phosphate, 50mM NaF, 0.5mM 2-ME (pH 8.0) using a NAP-5 column (GE Healthcare, Piscataway, NJ) and the concentration of protein adjusted to 5.8µM for wt Orf18 ArdA and Mut6 ArdA, 5.7µM for Mut5 ArdA and 5.6µM for L127E ArdA. All measurements were carried out at 20ºC using a cuvette with a 1 mm pathlength. The spectra show the characteristic negative peaks at 208 and 222 nm for α-helix secondary structures. DICHROWEB server analysis, http://dichroweb.cryst.bbk.ac.uk/html/home.shtml, indicated that all the proteins had an α-helix structure of 35-37%.

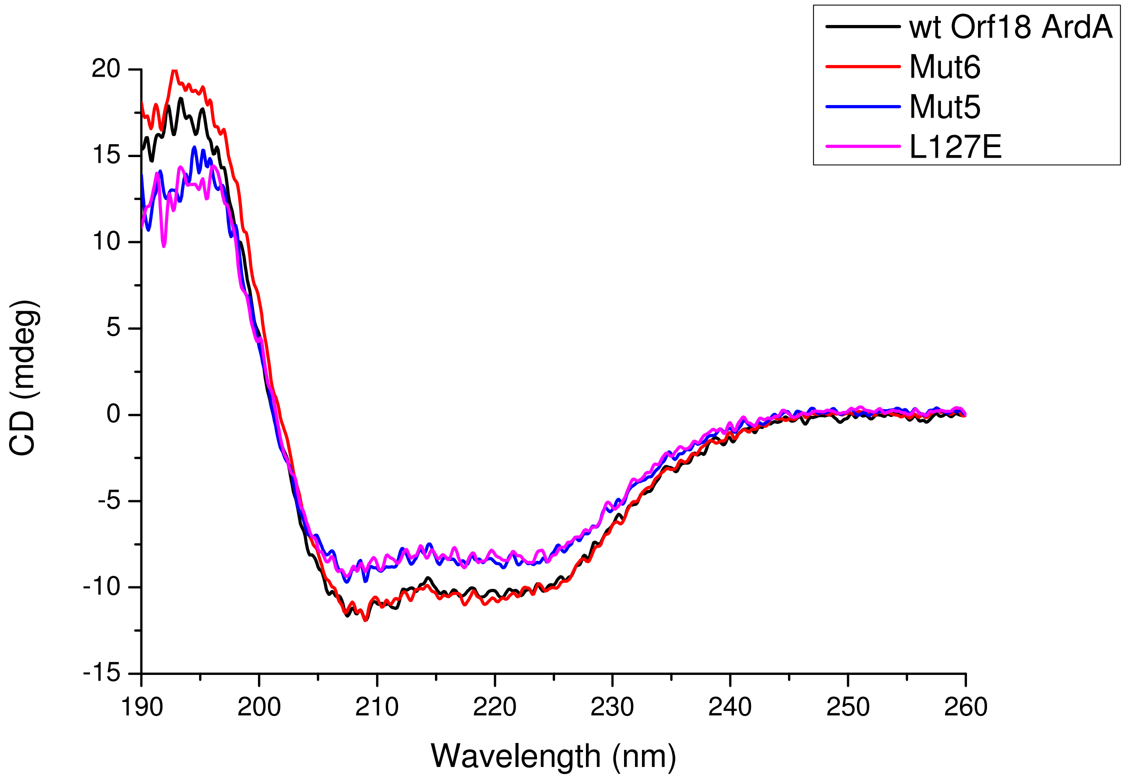

Supplementary Figure S4. **(a)** LC-MS analysis of the Mut5 ArdA yielded peaks of molecular mass 19121 and 19149 Da. The 28 Da mass difference is presumed to be due to the presence of a N-terminal formylated species, as has been observed with wild type ArdA [17]. The mass represents a deformylated species. The experimentally obtained isotopic peaks of the +11 charge state are shown overlaid with the theoretical expected apex for each isotopic peak, generated from the primary amino acid sequence of Mut5. The theoretical and experimental data overlap well. **(b)** LCMS analysis of Mut6. The experimental mass of Mut6 was found to be 19122 Da, which matched precisely with the theoretical value based on the primary amino acid sequence. A second peak was observed with a 28 Da increase in mass that corresponded to N-formylated methionine.

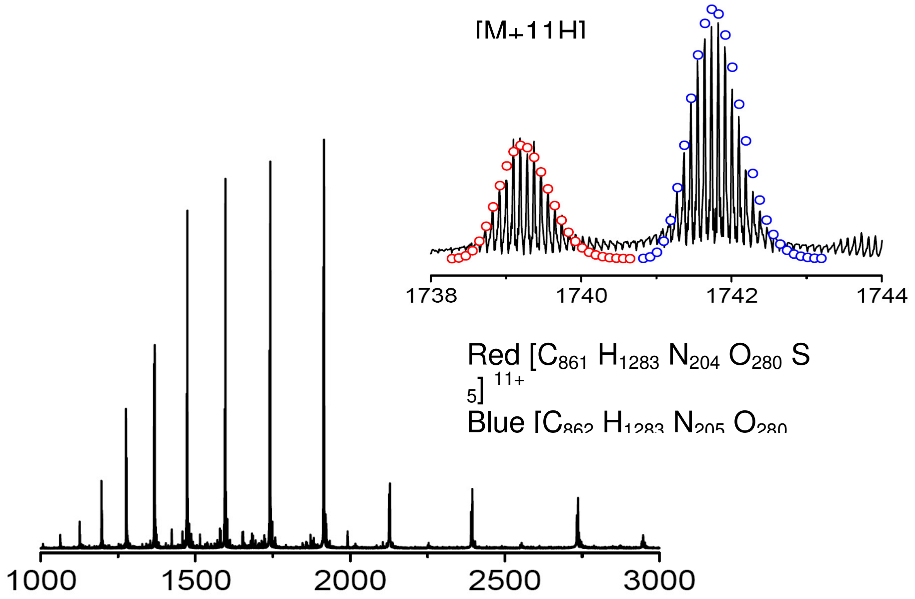

(a)

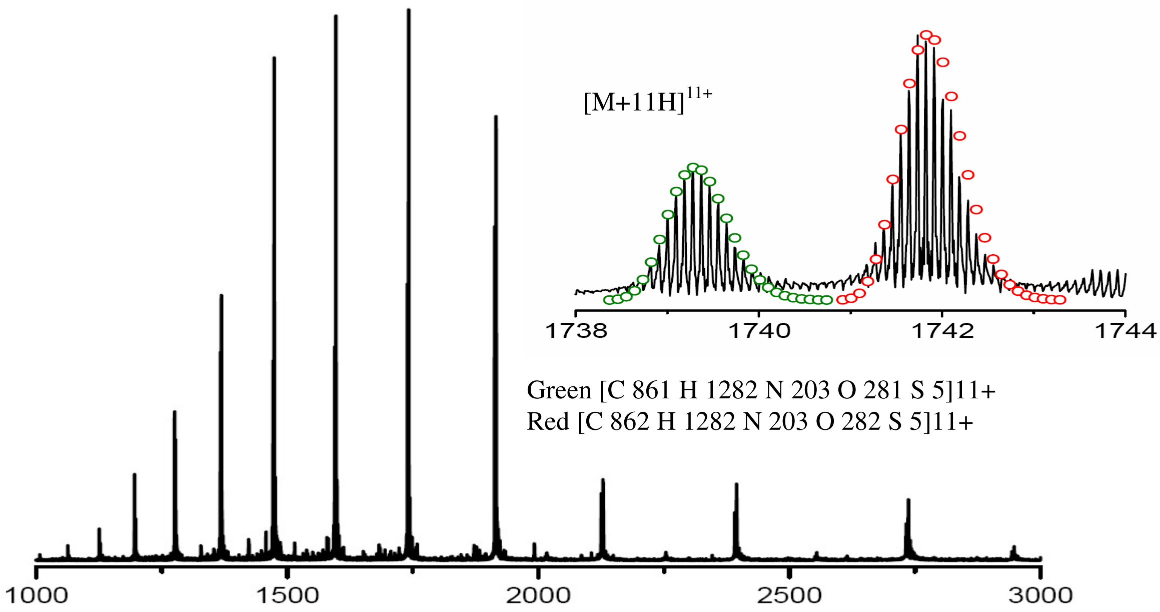

(b)

Supplementary Figure S5. (**a**) Size exclusion chromatography calibration curve using fluorescence detection with excitation at 295 nm and emission at 350 nm. The elution flow rate was 0.5 ml / min. β-Amylase: 200,000 Da. Alcohol Dehydrogenase: 150,000 Da. Albumin: 66,000 Da. Carbonic Anhydrase: 29,000 Da. Cytochrome C: 12,400 Da. (**b**) wt ArdA Orf18 elution profiles. (**c**) Mut5 ArdA elution profiles. (**d**) Mut6 ArdA elution profiles. (**e**) L127E ArdA elution profiles. The concentrations given in parts **b** to **e** are the injected protein monomer concentrations.

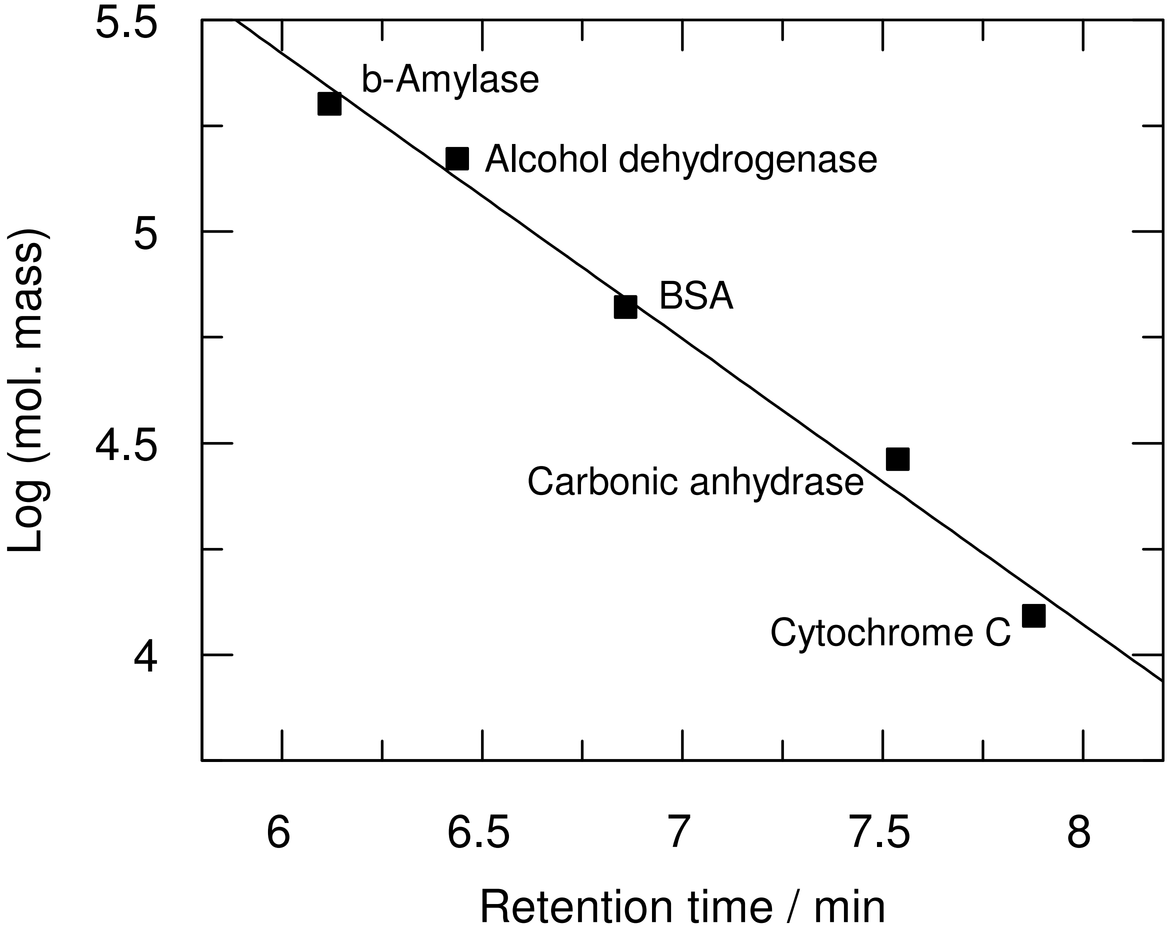

Linear Regression
Correlation Coefficient (r) = -0.9929

Variable	Value	Std. Err.
Intercept	9.4707	0.3263
Slope	-0.6749	0.0466

(**a**)

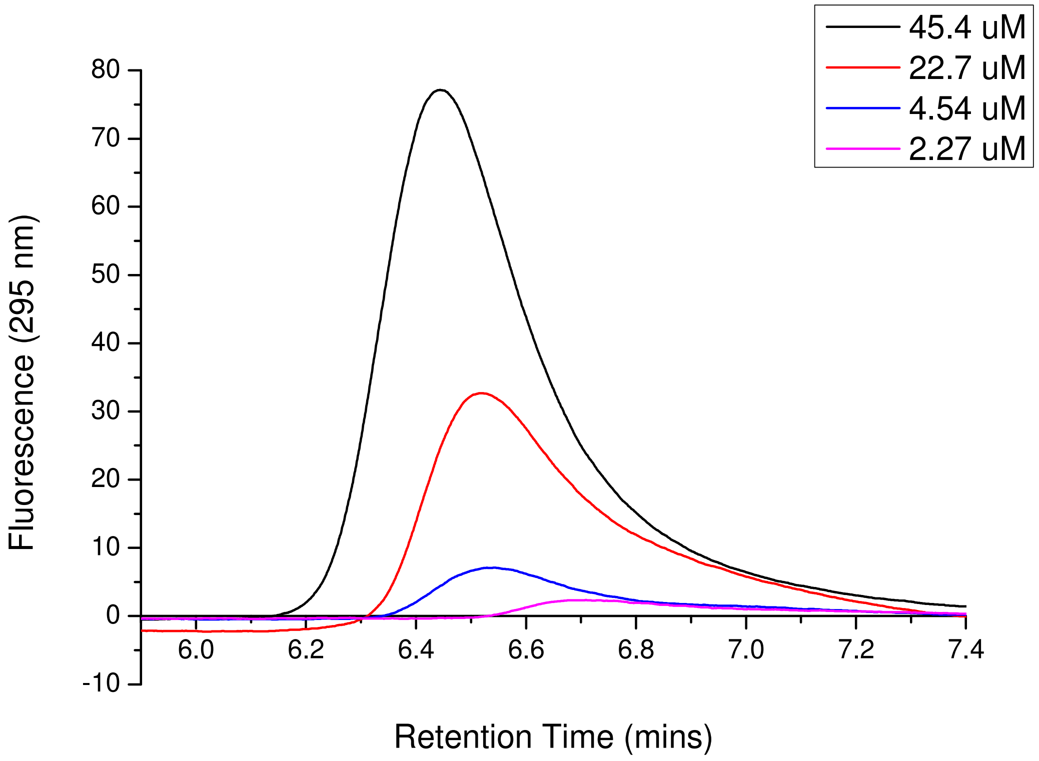
45.4 uM
22.7 uM
4.54 uM
2.27 uM
80
70
60
50
40
30
20
10
0
-10
Fluorescence (295 nm)
6.0
6.2
6.4
6.6
6.8
7.0
7.2
7.4
Retention Time (mins)

(b)

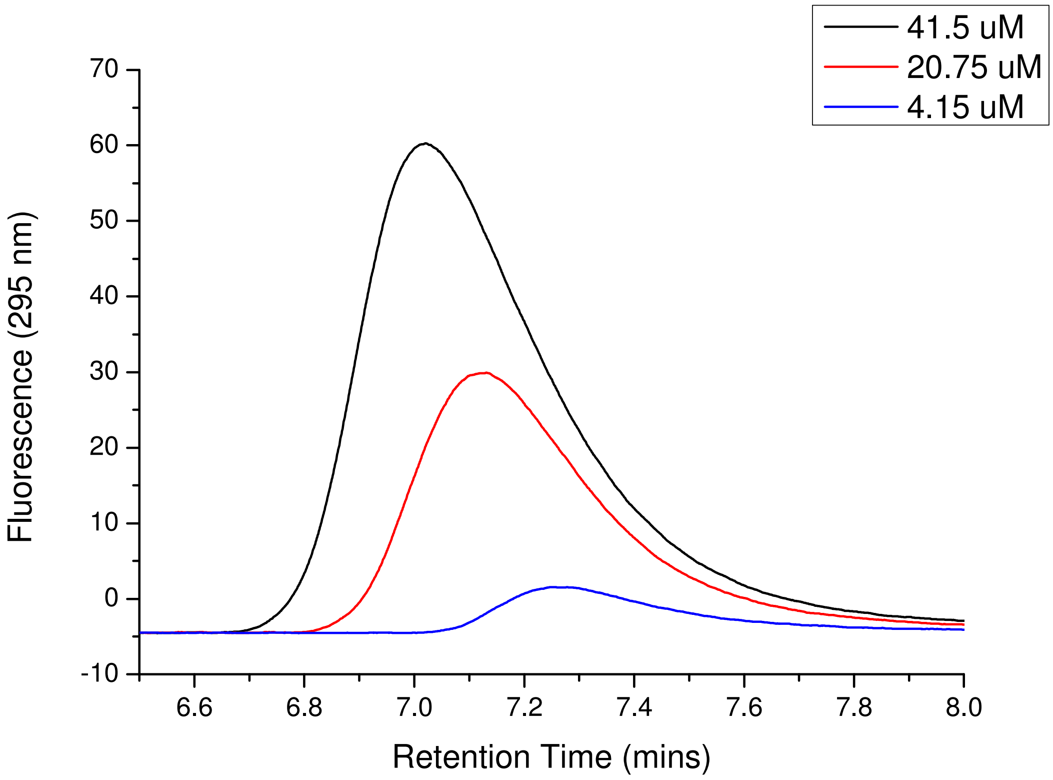
41.5 uM
20.75 uM
4.15 uM
70
60
50
40
30
20
10
0
-10
Fluorescence (295 nm)
6.6
6.8
7.0
7.2
7.4
7.6
7.8
8.0
Retention Time (mins)

(c)

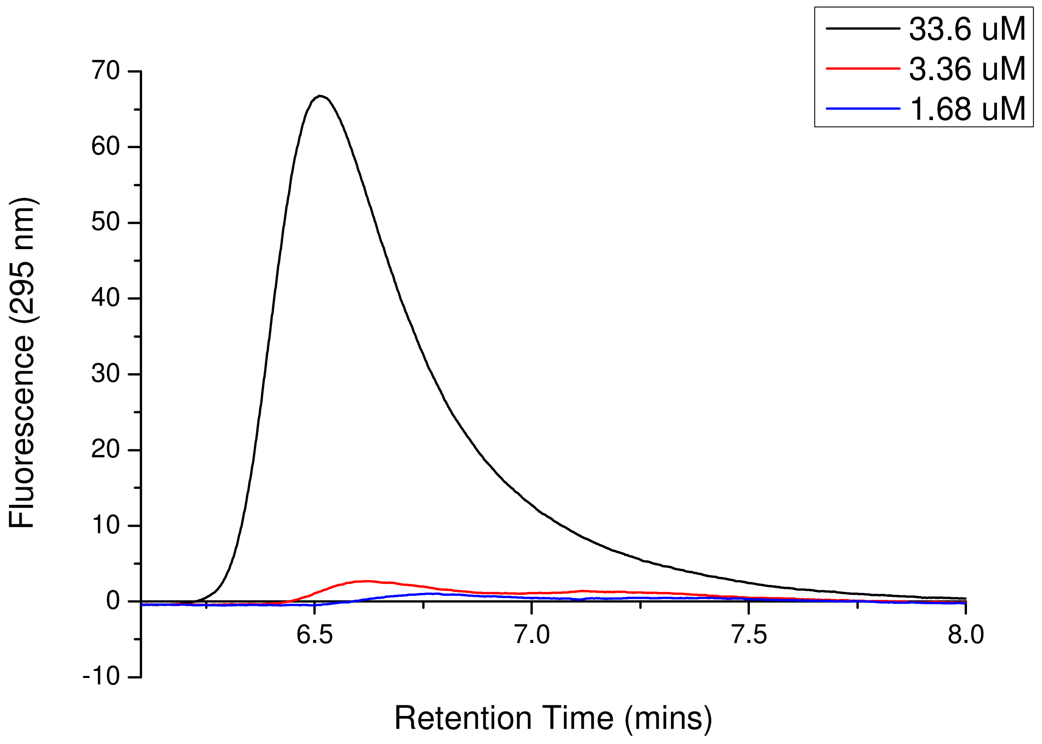
33.6 uM
3.36 uM
1.68 uM
Fluorescence (295 nm)
70
60
50
40
30
20
10
0
-10
6.5
7.0
7.5
8.0
Retention Time (mins)

(d)

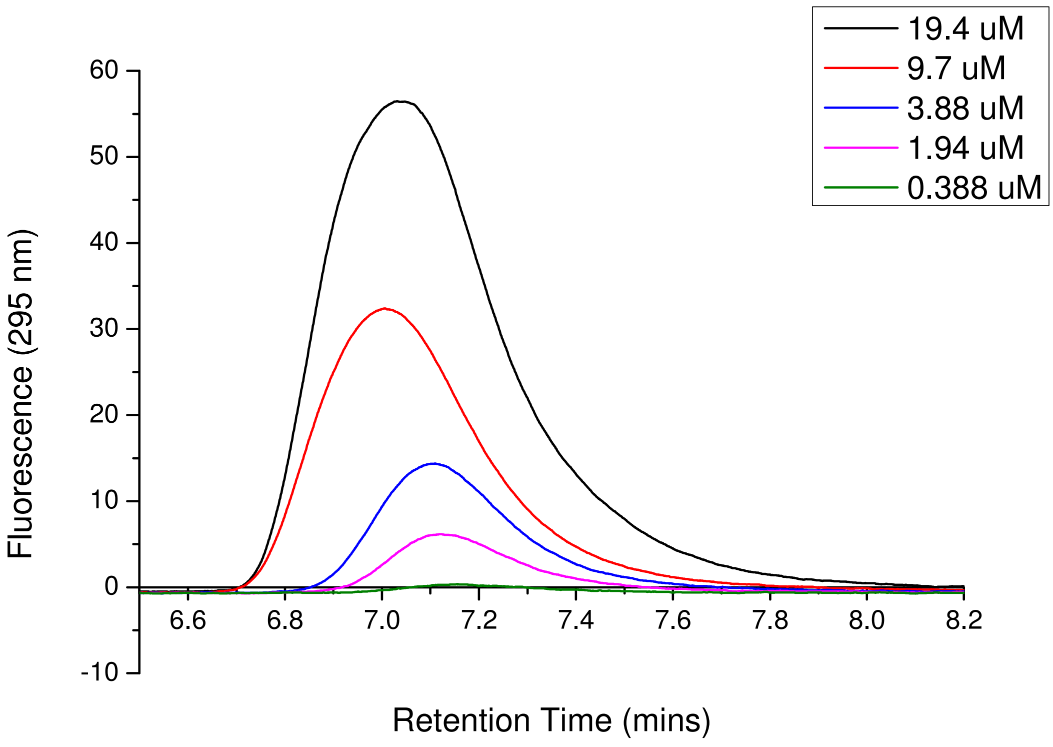
19.4 uM
9.7 uM
3.88 uM
1.94 uM
0.388 uM
Fluorescence (295 nm)
60
50
40
30
20
10
0
-10
6.6
6.8
7.0
7.2
7.4
7.6
7.8
8.0
8.2
Retention Time (mins)

(e)

Supplementary Figure S6. Selected sedimentation velocity scans showing data points (dots) and fitted data curves (lines) for every 3rd scan. Radial absorbance scans at 260nm were recorded for each cell over 15 hours. The scans are shown with dark green showing the earliest scan to light green showing the latest. **(a)** Raw data and fit scans for wt ArdA. **(b)** Raw data and fit scans for L127E ArdA.

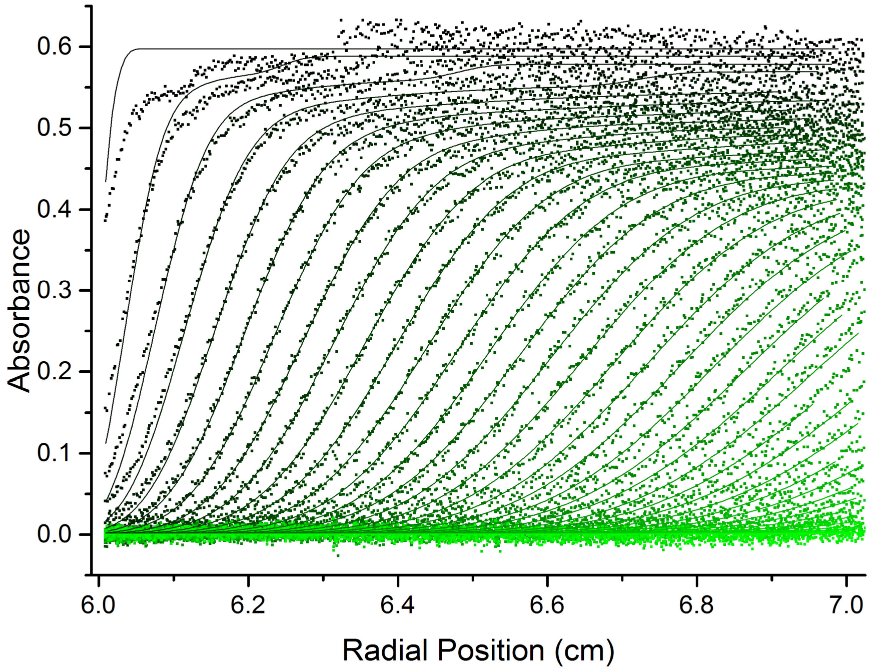

(a)

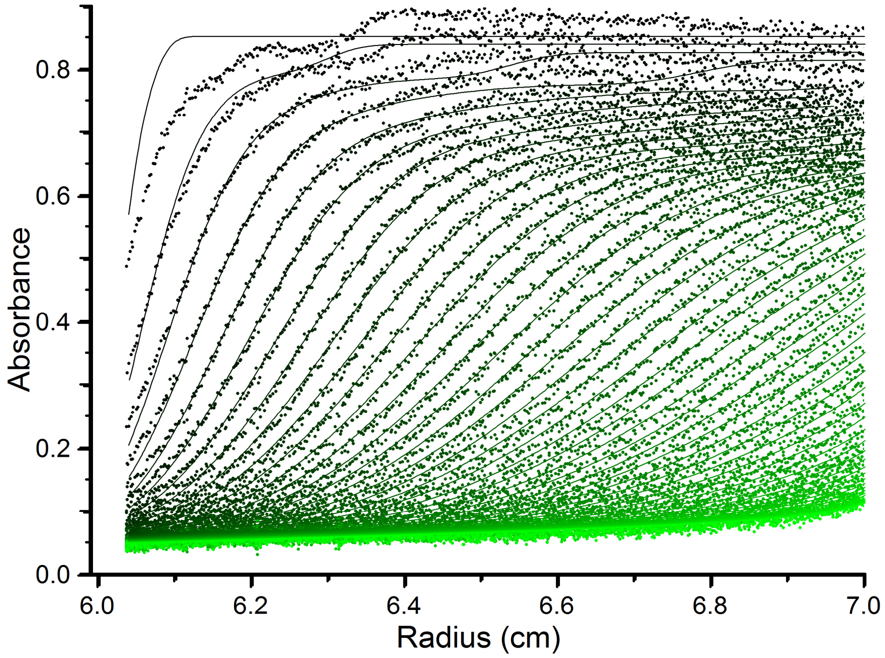

(b)
